# Structure and function of the peroxisomal ubiquitin ligase complex

**DOI:** 10.1042/BST20221393

**Published:** 2022-11-24

**Authors:** Peiqiang Feng, Michael L. Skowyra, Tom A. Rapoport

**Affiliations:** Howard Hughes Medical Institute and Department of Cell Biology, Harvard Medical School, 240 Longwood Avenue, Boston, Massachusetts 02115, U.S.A.

**Keywords:** peroxisomes, protein translocation, ubiquitin ligase

## Abstract

Peroxisomes are membrane-bounded organelles that exist in most eukaryotic cells and are involved in the oxidation of fatty acids and the destruction of reactive oxygen species. Depending on the organism, they house additional metabolic reactions that range from glycolysis in parasitic protozoa to the production of ether lipids in animals and antibiotics in fungi. The importance of peroxisomes for human health is revealed by various disorders — notably the Zellweger spectrum — that are caused by defects in peroxisome biogenesis and are often fatal. Most peroxisomal metabolic enzymes reside in the lumen, but are synthesized in the cytosol and imported into the organelle by mobile receptors. The receptors accompany cargo all the way into the lumen and must return to the cytosol to start a new import cycle. Recycling requires receptor monoubiquitination by a membrane-embedded ubiquitin ligase complex composed of three RING finger (RF) domain-containing proteins: PEX2, PEX10, and PEX12. A recent cryo-electron microscopy (cryo-EM) structure of the complex reveals its function as a retro-translocation channel for peroxisomal import receptors. Each subunit of the complex contributes five transmembrane segments that assemble into an open channel. The N terminus of a receptor likely inserts into the pore from the lumenal side, and is then monoubiquitinated by one of the RFs to enable extraction into the cytosol. If recycling is compromised, receptors are polyubiquitinated by the concerted action of the other two RFs and ultimately degraded. The new data provide mechanistic insight into a crucial step of peroxisomal protein import.

## Overview of protein import into peroxisomes

Peroxisomes are spherical organelles that occur in nearly all eukaryotic cells and are enclosed by a single lipid bilayer. They are almost universally involved in the oxidation of fatty acids and other biomolecules [1–9]. Many of these reactions generate hydrogen peroxide and other reactive oxygen species, so peroxisomes almost always also house catalase and other enzymes involved in redox homeostasis [10,11]. The metabolic functions of peroxisomes are surprisingly diverse in different organisms. For example, specialized peroxisomes called glyoxysomes in plants and filamentous fungi contain enzymes of the glyoxylate cycle [3,4]. In filamentous fungi, peroxisomes also produce β-lactam antibiotics such as penicillin [5], and specialized derivatives called Woronin bodies contain proteins that promote cell integrity and wound healing [12]. Glycosomes in kinetoplastid human parasites (e.g. *Leishmania* and trypanosomes) lack catalase but contain glycolytic enzymes [13]. In humans, peroxisomes notably house enzymes required for the synthesis of plasmalogens in the nervous system and of bile salts in the liver [7–9,14,15].

Most peroxisomal metabolic enzymes reside in the lumen (otherwise known as the matrix), but are synthesized in the cytosol and must be imported post-translationally into the organelle [1,2]. In humans, defects in peroxisomal matrix protein import cause peroxisome biogenesis disorders (PBDs), which frequently arise from inherited mutations in proteins called peroxins (PEX). The most severe mutations result in Zellweger syndrome that impairs multiple metabolic pathways and is often fatal [16,17].

**Figure 1.**
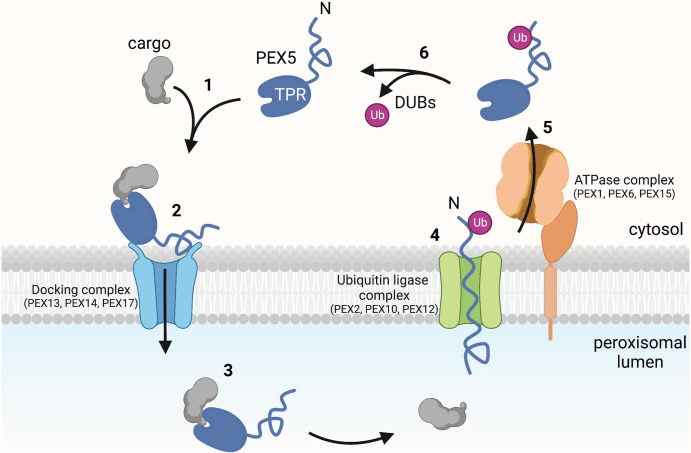
Model of peroxisomal matrix protein import. The scheme shows different steps during peroxisomal matrix protein import by the receptor PEX5. In step 1, the C-terminal SKL sequence of cargo is recognized and bound by the TPR domain of PEX5. In step 2, the complex of cargo and PEX5 binds to the docking complex in the peroxisomal membrane. In step 3, the PEX5-cargo complex moves all the way into the lumen. In step 4, the N terminus of PEX5 inserts into the pore of the ubiquitin ligase complex and a ubiquitin (Ub) molecule is attached. In step 5, monoubiquitinated PEX5 is pulled into the cytosol by the ATPase complex, resulting in the unfolding of PEX5 and cargo release. In step 6, ubiquitin is removed from monoubiquitinated PEX5 by deubiquitinating enzymes (DUBs).

Most matrix proteins contain a type 1 peroxisome targeting signal (PTS1) at their C terminus, which consists of the amino acid sequence Ser–Lys–Leu (SKL) or variants of it. This signal is recognized by the soluble receptor PEX5 in the cytosol [1,2] (Figure 1, step 1). PEX5 contains a C-terminal globular tetratricopeptide repeat (TPR) domain that directly binds the PTS1. PEX5 also contains a long unstructured region and a conserved cysteine residue near its N terminus. Cargo-laden PEX5 binds to a docking complex in the peroxisomal membrane, which consists of the conserved membrane proteins PEX13 and PEX14 [18,19] (Figure 1, step 2). In yeast, the docking complex also contains PEX17 [20]. Docking is followed by the complete translocation of cargo-bound PEX5 into the peroxisomal lumen [21,22] (Figure 1, step 3). This step remains mysterious, particularly because folded or even oligomeric proteins can traverse the membrane [23,24].

To start a new import cycle, PEX5 must return to the cytosol. This recycling step requires monoubiquitination of PEX5 at its conserved cysteine by a membrane-embedded, RING-type ubiquitin ligase complex composed of PEX2, PEX10, and PEX12 [1,2,25] (Figure 1, step 4). Ubiquitination of a cysteine residue in a substrate is unusual, as the formation of a ubiquitin thioester bond is mostly found as a transient modification in ubiquitin-activating and ubiquitin-conjugating enzymes [26]. Monoubiquitinated PEX5 is thought to be extracted from peroxisomes by a hexameric AAA ATPase formed from alternating copies of PEX1 and PEX6 [27] (Figure 1, step 5). The ATPase complex is anchored to the membrane by PEX15 in yeast and by PEX26 in mammals [28,29]. Finally, the ubiquitin moiety is removed in the cytosol by deubiquitinating enzymes (DUBs) (Figure 1, step 6), allowing PEX5 to initiate a new round of cargo translocation [1,30,31].

Some matrix proteins contain an alternative N-terminal peroxisome targeting signal called PTS2, whose recognition requires the adapter PEX7 [32–35]. In humans and plants, PEX7 binds to PEX5 through a motif in the receptor's unstructured N-terminal region [36,37]. In fungi, PEX7 associates instead with specialized receptors that include PEX18 and PEX21 in *Saccharomyces cerevisiae* [38–40], and PEX20 in other ascomycetous yeasts [41–43]. These receptors lack a TPR domain but are otherwise similar to PEX5: they all contain an unstructured N terminus [44]; are monoubiquitinated at a conserved N-terminal cysteine by the PEX2–PEX10–PEX12 ubiquitin ligase complex [45,46]; and require the PEX1–PEX6 ATPase to return to the cytosol [41].^1^
^1^PEX18, PEX20, and PEX21 have traditionally been called co-receptors. Given their well-established sequence and functional similarity to PEX5, however, we propose to regard all of these proteins as receptors. PEX18, PEX20, and PEX21 can only bind cargo in the presence of PEX7. Because of this property, PEX7 has traditionally been called a receptor. However, it is clear that PEX7 is unrelated to PEX5; cannot import cargo on its own; and uses a different mechanism to return to the cytosol. We therefore propose to call PEX7 a cargo adapter instead.

## The peroxisomal ubiquitin ligase complex

The components of the peroxisomal ubiquitin ligase complex were discovered individually in genetic screens in yeast, and by searching for genes that complement mammalian cells bearing Zellweger syndrome mutations [47–50]. The three discovered proteins (i.e. PEX2, PEX10, and PEX12) are conserved in all organisms known to harbor peroxisomes [51]. All three proteins constitutively associate with one another [52], and the stability of each component depends on the presence of the other members of the complex [53]. Recent purification experiments show that the three proteins indeed form a 1 : 1 : 1 stoichiometric complex in different fungi and likely in all other organisms [25]. Each of them notably contains a C-terminal RING finger (RF) domain that is a hallmark of many ubiquitin ligases [25,54].

The presence of three RF proteins in the same complex is unusual. Although RING-between-RING (RBR) ligases also contain three RFs, the RF domains are encoded in a single polypeptide chain [55]. The recently reported GATOR2 complex contains three distinct RF-containing subunits, but they probably perform structural roles and do not catalyze ubiquitination [56]. The role of the three RFs in the peroxisomal ubiquitin ligase complex has been unclear. The RF of PEX12 (i.e. RF12) has been implicated in monoubiquitination of import receptors [57], but more recent experiments indicate that this role is played by the RF of PEX2 (i.e. RF2) [25].

Like all ubiquitin ligases, the peroxisomal ubiquitin ligase complex needs to cooperate with ubiquitin-conjugating (E2) enzymes. In yeast, monoubiquitination requires the E2 enzyme PEX4 along with its membrane anchor and activator PEX22 [58]. These components are thought to be absent from higher organisms [51], where they may have been supplanted by more promiscuous E2 enzymes [59]. It is unclear which of the three RFs interacts with PEX4. Furthermore, as discussed below, additional E2 enzymes cooperate with the ligase complex.

PEX2, PEX10, and PEX12 are membrane-embedded proteins. Outside of their RING fingers, they do not share obvious sequence similarity, and until recently, it was thought that each contains only one or two transmembrane (TM) segments [60]. The exact mechanism by which the ligase complex facilitates receptor recycling into the cytosol has also remained elusive. Specifically, it has been unclear whether the ligase complex only catalyzes ubiquitination of the receptors or also facilitates their retro-translocation into the cytosol. A recent cryo-EM structure reveals that the ligase complex indeed forms a channel that likely mediates retro-translocation [25].

## The ubiquitin ligase complex has a constitutively open channel

**Figure 2.**
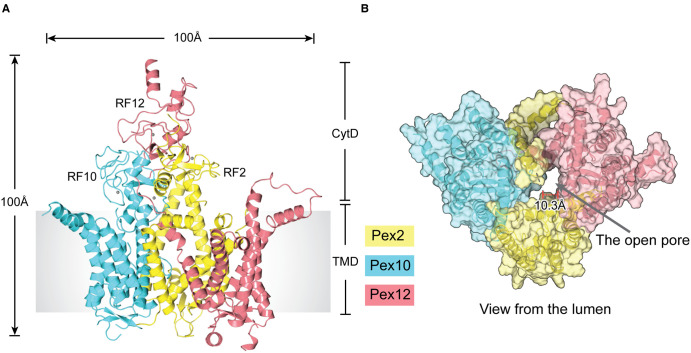
Structure of the peroxisomal ubiquitin ligase complex. (**A**) Cartoon model of the structure of the ubiquitin ligase complex from *T. thermophilus*, determined by cryo-EM. Shown is a side view, with the three components PEX2, PEX10, and PEX12 in different colors. The ligase complex has two distinct domains: a membrane-embedded domain (TMD) and a cytosolic domain (CytD) formed by the three RING fingers (RFs). CytD is also referred to as the RING finger tower. (**B**) View of the ligase complex from the lumen. Shown are cartoon models of the components together with semi-transparent space-filling models. Note the open pore. The broken line indicates a short luminal loop that is invisible in the density map, but would partially obstruct the pore.

The cryo-EM structure of the ligase complex from the thermophilic fungus *Thermothelomyces thermophilus* was determined at 3.1 Å overall resolution with the help of a selected Fab fragment [25]. The structure demonstrates that the ligase complex has two distinct domains: a membrane-embedded portion and a cytosolic RF tower (Figure 2A). The RF tower comprises the membrane-proximal RF2 and RF10 domains and the more distal RF12 (Figure 2A). The amino acid sequence of each of the three components (PEX2, PEX10, and PEX12) is conserved across eukaryotes, indicating that the structure is representative of peroxisomal ubiquitin ligase complexes in all organisms. Notably, point mutations that cause disease in humans map to the TM segments as well as to the RFs.

Contrary to previous assumptions, each subunit of the complex has five TM segments. Four of these form superimposable structures, indicating that, despite their sequence differences, the three proteins have a common ancestor. The TM segments generate a channel with an open pore ∼10 Å in diameter (Figure 2B). The channel seems to have rigid walls, so it unlikely undergoes a conformational change into a closed state. There are also no polypeptide segments that could plug the pore; the N-terminal tail of PEX12 resides near the pore on the lumenal side, but it is not essential for function. Thus, the ligase pore seems to be constitutively open.

The open pore implies that the peroxisomal membrane might be permeable to small molecules. The actual permeability of peroxisomes has been a controversial issue. For example, some reports suggest that the pH is the same inside and outside of the organelle [61], whereas others claim differences [62,63]. Furthermore, certain small molecules such as ATP and NADH may require specific transporters to enter peroxisomes [64]. However, the size of the ligase pore is consistent with the reported permeability of the peroxisomal membrane to molecules smaller than ∼800 Da [65,66]. If the peroxisomal membrane is porous, it might allow intermediates of lumenal metabolic pathways to escape into the cytosol, but perhaps the high concentration of lumenal enzymes causes ‘metabolic channeling’, such that the product of one enzyme is immediately transferred to the next enzyme. The main function of peroxisomes may thus be to concentrate sequential enzymes, with distinct metabolic reactions concentrated in different organisms. The existence of membrane pores that allow the passive diffusion of small molecules would be unusual, shared only with the outer membranes of mitochondria, chloroplasts, and bacteria.

## The ubiquitin ligase complex functions as a retro-translocon

The open pore of the ligase complex may also provide the translocation path for recycling peroxisomal import receptors. Because PEX5 moves with cargo completely into the lumen [21], and because the ligase complex has no lateral gate [25], the receptors can only access the catalytic RFs from the peroxisomal lumen rather than sideways through the membrane. The N-terminal segment of the receptors would insert into the pore, positioning the conserved cysteine residue close to one of the RFs. Insertion of the N terminus may be initiated by an interaction of a conserved amphipathic helix in the receptor with the ligase complex [21].

RF2 sits right above the pore [25], suggesting that RF2 is responsible for receptor monoubiquitination at the conserved cysteine. However, monoubiquitination of PEX5 by RF2 has not yet been recapitulated with purified components, perhaps because the thioester-linked monoubiquitinated PEX5 is too labile or transient. While PEX5 monoubiquitination has been reported to occur with purified rat liver peroxisomes [59,67], the role of RF2 in this system has not been investigated. The other two RFs (RF10 and RF12) are more distant and cannot be reached by a polypeptide located inside the pore, as the path is sterically blocked by a loop of PEX10. As discussed below, RF10 and RF12 are involved in an alternative ubiquitination pathway.

Consistent with the proposed translocation path for recycling receptors, the function of the ligase complex is compromised by mutations that reduce its pore size [25]. Furthermore, in a cell-free system based on *Xenopus* egg extract, a prominent retro-translocation intermediate of PEX5 is observed, in which PEX5 is associated with the ligase complex and ∼20–30 N-terminal residues have emerged into the cytosol [21]. The narrow pore would explain why the C-terminal TPR domain is unfolded when the PEX1–PEX6 ATPase pulls monoubiquitinated PEX5 into the cytosol [21]. Unfolding the TPR domain provides a simple mechanism of cargo release in the lumen of peroxisomes. Despite this recent progress, direct evidence for the receptor being located inside the pore is still missing.

PEX5 export out of peroxisomes resembles ER-associated protein degradation (ERAD) [25,68]. In ERAD, multi-spanning ubiquitin ligases provide a passageway for misfolded ER proteins through the membrane [69]. Once on the cytosolic side, these proteins are ubiquitinated by the ubiquitin ligase and extracted from the membrane by the p97/CDC48 AAA ATPase [70]. Similarly, retro-translocation of PEX5 is mediated by a multi-spanning ubiquitin ligase complex, followed by receptor ubiquitination in the cytosol by the ubiquitin ligase complex and extraction by the PEX1–PEX6 AAA ATPase.

## Why does the ligase complex have three RING fingers?

Previous studies raised the possibility that the peroxisomal ubiquitin ligase complex may also polyubiquitinate import receptors. When the normal recycling of receptors is blocked, for example, by inactivating the PEX1–PEX6 ATPase, the receptors are instead polyubiquitinated on lysines and subsequently degraded by the proteasome [24]. This alternative pathway has been termed ‘Receptor Accumulation and Degradation in the Absence of Recycling,' or simply RADAR [24,41]. Indeed, RF10 and RF12 seem to be involved in polyubiquitination, cooperating with the E2 enzymes Ubc4 and Ubc5 [71]. RF10 is a canonical RF and has some polyubiquitination activity on its own, but it is greatly stimulated by RF12 [25,53]. On the other hand, RF12 lacks the structural features required for interaction with an E2 enzyme and is inactive by itself [25]. RF12 therefore seems to be an activator of RF10. The RF10–RF12 heterodimer structurally resembles homodimers of other ubiquitin ligases, and mutations based on this similarity have the predicted detrimental effects on their polyubiquitination activity [25].

**Figure 3.**
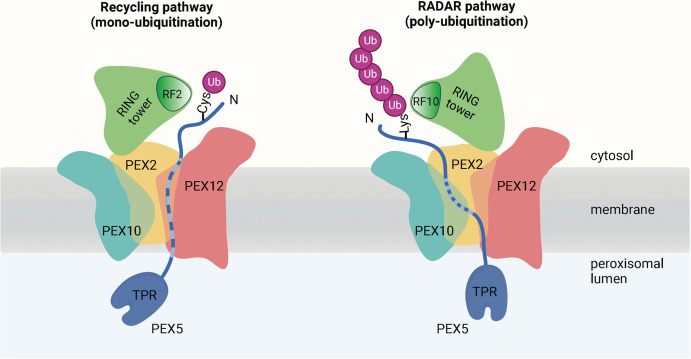
Model for PEX5 mono- and polyubiquitination by the peroxisomal ubiquitin ligase complex. In the normal receptor recycling pathway (left), the receptor PEX5 inserts an N-terminal segment into the ligase pore, positioning a conserved cysteine for monoubiquitination by the RING finger of PEX2 (RF2). A region of PEX5 is in the background behind PEX12 and is indicated by a broken line. In the RADAR pathway (right), which is activated when normal recycling is compromised, the N-terminal segment of PEX5 moves sideways to position a lysine residue for polyubiquitination by RF10. A region of PEX5 is in the background behind PEX10 and is indicated by a broken line.

Mutations designed to abolish the interaction of RF2 or RF10 with their E2 enzymes have little effect on their own on peroxisomal matrix protein import in *S. cerevisiae* [25]. However, a double mutant in both RF2 and RF10 showed a significant import defect, suggesting that the polyubiquitination pathway can compensate for monoubiquitination during normal receptor recycling. This model is supported by PEX5 overexpression experiments in wild-type cells. Expression of PEX5 mutants lacking either the conserved cysteine for monoubiquitination, or the lysine residues for polyubiquitination, did not cause an import defect. When both mutations were combined, however, import was precluded, likely because this mutant cannot be removed from the ligase pore and thereby prevents the export of endogenous PEX5 molecules. Similarly, peroxisomal protein import was reduced when the cysteine mutant was overexpressed in cells with a polyubiquitination-defective RF10 mutant, or when the lysine mutant was overexpressed in a RF2 mutant with impaired E2 interaction. Taken together, these results support a model in which RF2 catalyzes monoubiquitination during normal receptor recycling, whereas RF10 and RF12 function in an alternative polyubiquitination pathway (Figure 3). A polypeptide located inside the ligase pore would have to move sideways to reach the active site of RF10 [25]. This lateral movement would only be possible if a PEX10 loop is displaced. The sequence of the loop is poorly conserved and its deletion does not affect protein import; in higher organisms and some fungi, it seems to be shorter or even absent.

In the cryo-EM structure, the predicted interactions of RF2 and RF10 with E2 enzymes would lead to steric clashes [25], indicating that both RFs need to change their positions to become active. Because RF2 is only weakly associated with the other RFs, it could dissociate and rotate to allow binding of the PEX4∼ubiquitin adduct. RF10 and RF12 would have to tilt as a unit to allow E2 binding. RF2 and RF10 are connected by flexible loops with the membrane-embedded regions, so these conformational changes would not affect the pore. How exactly RF2 and RF10 would catalyze mono- and polyubiquitination, respectively, requires structures of the ligase complex with the cooperating E2 enzymes and PEX5.

## Perspectives

- Peroxisomal matrix protein import remains an important research topic, particularly because many aspects of this process differ from the better known translocation pathways into the endoplasmic reticulum (ER) or mitochondria. The field also has important implications for our understanding of peroxisome biogenesis disorders and might lead to treatment of these devastating diseases.
- The cryo-EM structure of the peroxisomal ubiquitin ligase complex, coupled with biochemical and *in vivo* experiments in yeast and in a novel *Xenopus* egg extract cell-free system, have significantly advanced our knowledge of peroxisomal matrix protein import. These results have led to an updated model for how PEX5 translocates into and out of peroxisomes to drive matrix protein import (Figure 1). It is now clear that PEX5 accompanies cargo completely into the lumen, and is then returned to the cytosol by retro-translocation through the pore of the peroxisomal ubiquitin ligase complex. Extraction of PEX5 from the lumen results in unfolding of the receptor and cargo release inside the organelle. Import and export of PEX5 are probably coupled, as import is likely driven by the energy required for export. In fact, it is possible that PEX5's N terminus is exported before the C-terminal TPR domain is fully translocated into the lumen. The proposed model could also explain how PTS2 cargo is imported. The complex of PTS2 cargo, receptor, and PEX7 would move into the lumen [33–35], and subsequent export of the receptor through the ligase pore would strip off both the cargo and PEX7. How PEX7 returns to the cytosol remains to be clarified, as it lacks an unstructured region for insertion into the ligase pore and a cysteine for monoubiquitination [32].
- Despite the progress, many aspects of peroxisomal protein import remain unclear. Arguably the most mysterious remains the mechanism by which cargo-bound PEX5 is translocated from the cytosol into the lumen. Which components form the putative import pore? How do folded and oligomeric proteins move across the membrane? In this respect, the mechanism of translocation must differ dramatically from that into the ER or mitochondria, which can only import proteins in an unfolded conformation [72,73]. Other unresolved questions concern the mechanisms by which PEX5 is monoubiquitinated and subsequently extracted by the PEX1–PEX6 ATPase. Although some low-resolution structures are available [27,28,74], the PEX1–PEX6 ATPase is much less studied than the related p97/CDC48 ATPase. The RADAR pathway also remains poorly understood, both with respect to the ATPase that extracts the polyubiquitinated receptors into the cytosol and its physiological significance. Proteomics experiments suggest that the polyubiquitylation pathway not only mediates the degradation of import receptors but also maintains the homeostasis of other import factors, such as docking protein components [25]. The pathway may also be involved in the degradation of several peroxisomal membrane proteins [25,75].
- The recent insight may also have implications for peroxisome biogenesis disorders. Mutations that solely affect the polyubiquitination pathway seem to cause a less severe phenotype than those disrupting the monoubiquitination/recycling pathway or the integrity of the ubiquitin ligase complex [76]. It might be possible to find small molecules that restore or stimulate polyubiquitination and thus correct these milder disease phenotypes.

## Abbreviations

cryo-EM: cryogenic electron microscopy
ER: endoplasmic reticulum
ERAD: ER-associated protein degradation
PBDs: peroxisome biogenesis disorders
PEX: peroxin
PTS: peroxisomal targeting signal
RADAR: receptor accumulation and degradation in the absence of recycling
RBR: RING-between-RING
RF: RING finger
RING: really interesting new gene
TM: transmembrane
TPR: tetratricopeptide repeat
